# Structural bioinformatics analysis of SARS-CoV-2 variants reveals higher hACE2 receptor binding affinity for Omicron B.1.1.529 spike RBD compared to wild type reference

**DOI:** 10.1038/s41598-022-18507-y

**Published:** 2022-08-25

**Authors:** Vedat Durmaz, Katharina Köchl, Andreas Krassnigg, Lena Parigger, Michael Hetmann, Amit Singh, Daniel Nutz, Alexander Korsunsky, Ursula Kahler, Centina König, Lee Chang, Marius Krebs, Riccardo Bassetto, Tea Pavkov-Keller, Verena Resch, Karl Gruber, Georg Steinkellner, Christian C. Gruber

**Affiliations:** 1 Innophore GmbH, 8010 Graz, Austria; 2 Institute of Molecular Biosciences, University of Graz, 8010 Graz, Austria; 3 Austrian Centre of Industrial Biotechnology, 8010 Graz, Austria; 4 AWS Diagnostic Development Initiative-Global Social Impact, Seattle, WA 98109 USA; 5 Amazon Web Services EMEA SARL, 80807 Muenchen, Germany; 6 Field of Excellence BioHealth–University of Graz, 8010 Graz, Austria

**Keywords:** Viral infection, Structural biology

## Abstract

To date, more than 263 million people have been infected with SARS-CoV-2 during the COVID-19 pandemic. In many countries, the global spread occurred in multiple pandemic waves characterized by the emergence of new SARS-CoV-2 variants. Here we report a sequence and structural-bioinformatics analysis to estimate the effects of amino acid substitutions on the affinity of the SARS-CoV-2 spike receptor binding domain (RBD) to the human receptor hACE2. This is done through qualitative electrostatics and hydrophobicity analysis as well as molecular dynamics simulations used to develop a high-precision empirical scoring function (ESF) closely related to the linear interaction energy method and calibrated on a large set of experimental binding energies. For the latest variant of concern (VOC), B.1.1.529 Omicron, our Halo difference point cloud studies reveal the largest impact on the RBD binding interface compared to all other VOC. Moreover, according to our ESF model, Omicron achieves a much higher ACE2 binding affinity than the wild type and, in particular, the highest among all VOCs except Alpha and thus requires special attention and monitoring.

## Introduction

In the COVID-19 pandemic, more than 263 million people have been infected with severe acute respiratory syndrome coronavirus 2 (SARS-CoV-2) as reported by WHO in December 2021^1^. So far, the global spread was distributed over several pandemic waves in many countries^2^: each wave was characterized by the emergence of SARS-CoV-2 variants with some becoming dominant in regional endemic outbreaks^3^ or on a global level^4–6^. Whereas the timeline of the waves and the distribution of variants may appear to be asynchronous, several variants emerged that significantly dominated the global pandemic event, namely the variants of concern (VOCs), Alpha, Beta, Gamma and Delta^7,8^.

While preparing potential drug targets^9^ of the SARS-CoV-2 proteome^10^ for our ultra-large-scale virtual drug screening^11,12^, we started monitoring the emerging diversity of the SARS-CoV-2 genome landscape on a structural bioinformatics level in order to keep design and strategy for the screening aligned with the actual pandemic situation. Recently, we reported on a then emerging variant carrying a particular amino acid exchange (S477N or S477G) in a highly flexible region of the spike receptor binding domain (RBD). We showed that this kind of exchange results in increased flexibility and increased affinity^13^ for the human receptor angiotensin-converting enzyme 2 (hACE2)^14,15^. It is reported elsewhere that an increased spike-hACE2 affinity correlates with higher infectiousness^16–18^.

Still, indications for higher affinity or infectiousness do not automatically carry over to the identification of dominant SARS-CoV-2 variants, due to the complex dynamics of a pandemic situation. For example, the notable missense single mutation S477N/G embedded in the variant under monitoring (VUM) B.1.526 Iota did not become a major variant and spread only locally, not globally. In particular, local outbreaks e.g. in New York City and New York State^19^ were characterized by domination of this variant, before the Delta variant became dominant there.

In the framework of our virus.watch project in cooperation with Amazon Web Services’ Diagnostic Development Initiative we continue the structural monitoring of emerging drug- and disease-relevant SARS-CoV-2 mutations. Overall, the average number of amino acid exchanges per sequence, collected over a large set of available sequences, has increased dramatically in the past year. We present a more detailed analysis below.

Following the path of the S477G/N mutation, the currently widely discussed VOC B.1.1.529 Omicron raised our interest shortly after its genome became available on November 8th, 2021. It also contains the S477N mutation, which is the first time that this particular amino acid exchange is present in an official VOC.

The emerging SARS-CoV-2 VOC Omicron was first reported during an endemic outbreak in Botswana and South Africa (hCoV-19/SouthAfrica/NICD-N21441/2021, GISAID ID EPI_ISL_6913995)^20^. It features a high number of mutations throughout the viral genome, 39 of which cause changes in the amino acid sequence of the spike protein^21–23^. Within the spike RBD there are 15 amino acid replacements including our previously characterized mutation S477N. Thus, VOC Omicron comprises a three times higher number of mutations compared to the next most varied RBD of VUM B.1.640^21^.

A structural bioinformatics analysis of this variant, in particular its expected binding mode and predicted affinity, is clearly needed. Such a claim is more than reasonable, both based on the extraordinary amount of RBD amino acid exchanges in this variant as well as due to the presence of single mutations known to increase the binding affinity.

Furthermore, since the spike protein is also recognized by the immune system as a primary antigenic target to neutralize the virus, a close inspection of relevant changes in the interaction pattern is crucial. This is true in particular for, e.g., deepening the understanding of biomolecular recognition of antibodies that is the basis for many of today's approved COVID-19 vaccines^24,25^. Concrete examples are Comirnaty (BNT162b2) by BioNTech SE, or Spikevax (mRNA-1273) by ModernaTX, Inc., who announced the development of an updated booster vaccine within the next few months. In the context of potential antibody evasion^26^, vaccine manufacturers rely also on structural insights and consider them highly relevant, e.g., BioNTech has stated to closely monitor the emerging genomic diversity^27^. In another line of application, several biotherapeutics currently in development, e.g., a recombinant human soluble hACE2-based decoy^28,29^, rely on an effective binding to the spike protein at the surface of the virion.

Herein, we report the results of our structural bioinformatics approach regarding the influence of these amino acid exchanges on the affinity of the spike RBD to the human receptor hACE2. Following our spike RBD sequence diversity studies and a visual analysis of related changes in electrostatics and hydrophobicity distributions, we developed and employed an empirical scoring function (ESF) closely related to the linear interaction energy (LIE) method that was calibrated on experimental binding data and molecular dynamics trajectories. Our analysis focuses on primary effects of observed amino acid exchanges. Alterations of the glycosylation pattern or effects on the oligomerization of spike proteins were not considered at this stage. This approach delivers an early structural insight and binding affinity estimates before experimental complex structures and experimental binding data will become available within the next months.

## Results

In order to enable a comprehensive structural analysis of emerging partial or complete SARS-CoV-2 genomes, we follow a three-phase approach. In the first phase, we analyze new genomes (e.g. from GISAID or sequences directly provided by associated laboratories) for sequences with amino acid exchanges in regions of SARS-CoV-2 protein structures that are potentially relevant for drug binding or cell uptake.

In phase two, we look for sequences where one or more mutations are found that have not yet been investigated in our structural analysis pipeline. In such a case, the sequence is submitted to a structure modeling workflow, whose results can be used to predict an influence caused by the respective amino acid exchanges on active site regions or cavities that are potentially relevant for drug development. In the case of the spike protein, on whose RBD we focus herein, relevant models are analyzed using our Catalophore Halo technology (defined and illustrated below).

In phase three, we compare any modified RBD’s Halo to the wild type Halo. If such a Halo comparison shows a substantial change, we expose the new variant to an ESF-based molecular dynamics modeling pipeline in order to predict the corresponding change in binding affinity.

### SARS-CoV-2 sequence analysis of spike-RBD diversity

By December 6th 2021, 5,799,116 SARS-CoV-2 genome sequences and 5,649,261 spike-protein sequences were available at GISAID^21^. For the sequence analysis of the SARS-CoV-2 RBD diversity we extracted 5,173,253 RBD sequences by filtering the spike protein sequences for the RBD flanking residues “TSNF” (position 315–318) and “NFNG” (position 542–545) as well as for correct size of 223 amino acids. Within these sequences we identified a total number of 7,700,325 amino acid exchanges, which amounts to 0.67% of all residues. Compared to August 2020, when 185 mutations were identified in 73,042 spike RBD sequences^13^, this occurrence per sequence of mutations shows a 588-fold increase, highlighting the progressive evolution of this virus^30,31^. The accumulation of mutations was significantly higher in the receptor binding motif (RBM), the 72-amino acid long sequence within the RBD, which mediates contacts with hACE2^15^: with a total of 7,451,124 amino acid exchanges, meaning 2.00% of all residues within all of the collected RBM sequences, the relative number of mutations within the RBM increased 1547-fold compared to August 2020, where 68 amino acid exchanges were found in 73,042 RBM sequences^13^.

**Figure 1:**
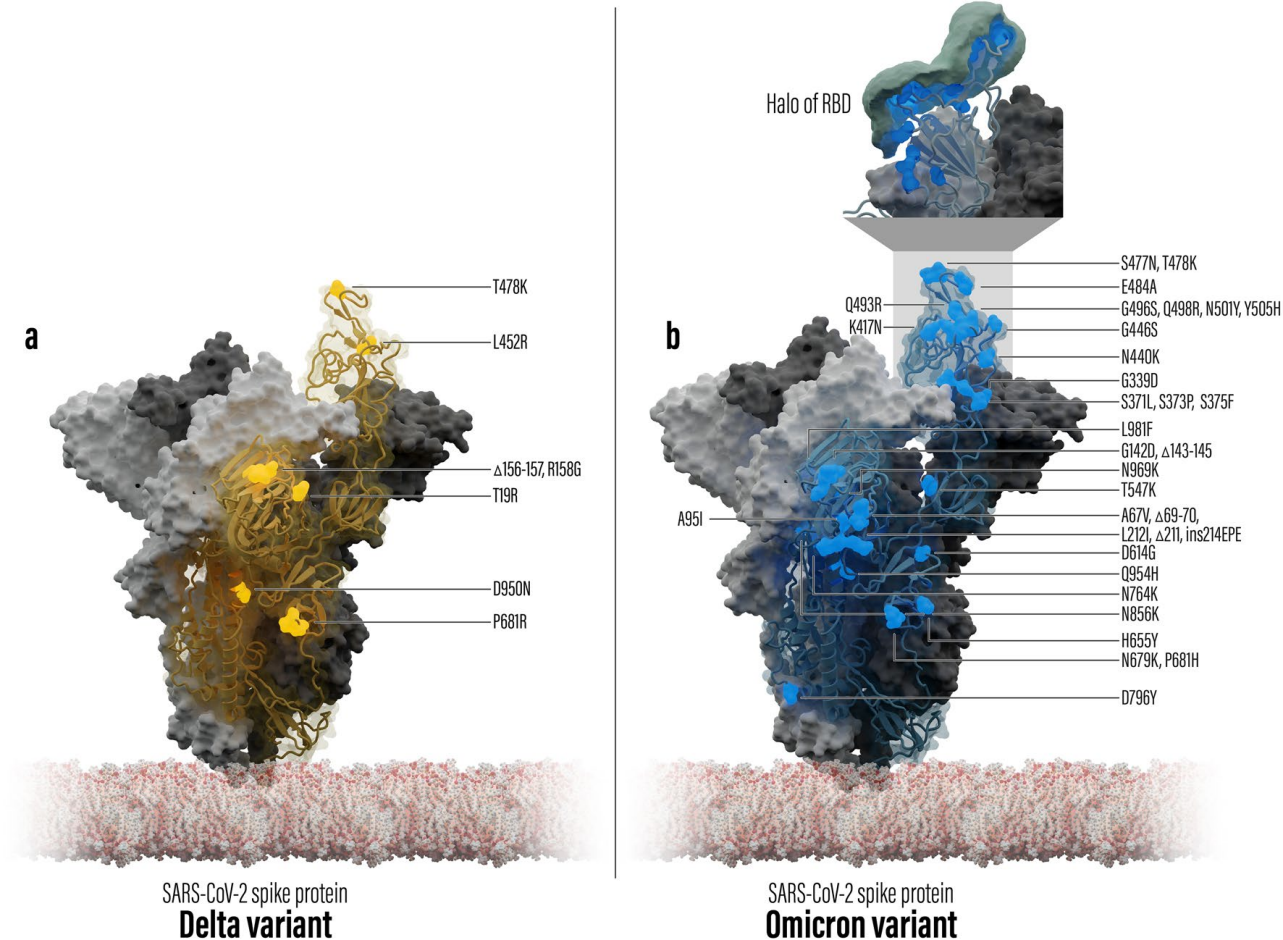
Overview of structural models of the spike protein of SARS-CoV-2 including mutation locations compared to the wild type. One of the trimer chains is shown as a transparent surface with the RBD domain on top of the spike protein. (**a**) Delta variant, mutation locations are shown as yellow spheres. (**b**) Omicron variant, mutation locations are shown as blue spheres. The top box shows a zoom to the RBD with the calculated Halo point cloud of the RBD.

Out of the 5,173,253 considered RBD and RBM sequences, 85.11 and 84.67%, respectively, contain at least one amino acid exchange, with 99.48% of all mutated RBDs bearing mutations within the RBM. The mean number of mutations within the RBD and RBM sequences in this analysis are 1.75 ± 0.57 and 1.70 ± 0.49, respectively. With 15 amino acid substitutions located in the RBD, ten of which occur in the RBM, the recently discovered VOC Omicron stands out from previous variants by an at least three times higher number of mutations in this region^21^ (see also Fig. 1b).

Referring to a list of 143 representative SARS-CoV-2 spike-protein sequences available at GISAID^21^, six of the amino acid exchanges within the Omicron RBD co-occur in other RBD variants, namely K417N (present in seven variants including VOC Beta), N440K (three variants), S477N (four variants), T478K (75 variants, including VOC Delta), E484A (two variants) and N501Y (33 variants including VOCs Alpha, Beta and Gamma). Their potential influences on one or more properties such as protein stability and flexibility as well as binding and sensitivity to antibodies have been evaluated, whereby exchanges N440K, S477N, T478K and N501Y were reported to possibly enhance binding affinity to hACE2^13,32–36^.

The remaining nine mutations within the Omicron RBD (G339D, S371L, S373P, S375F, G446S, Q493R, G496S, Q498R and Y505H) do not occur in any of the 143 representative SARS-CoV-2 spike protein sequences^21^. Nevertheless, possible influences of these amino acid exchanges have already been studied in silico or in vitro, with G339D, Q493R and Q498R being predicted to enhance binding to hACE2^35,37,38^.

### Visualization of amino acid exchanges using difference Halos

**Figure 2:**
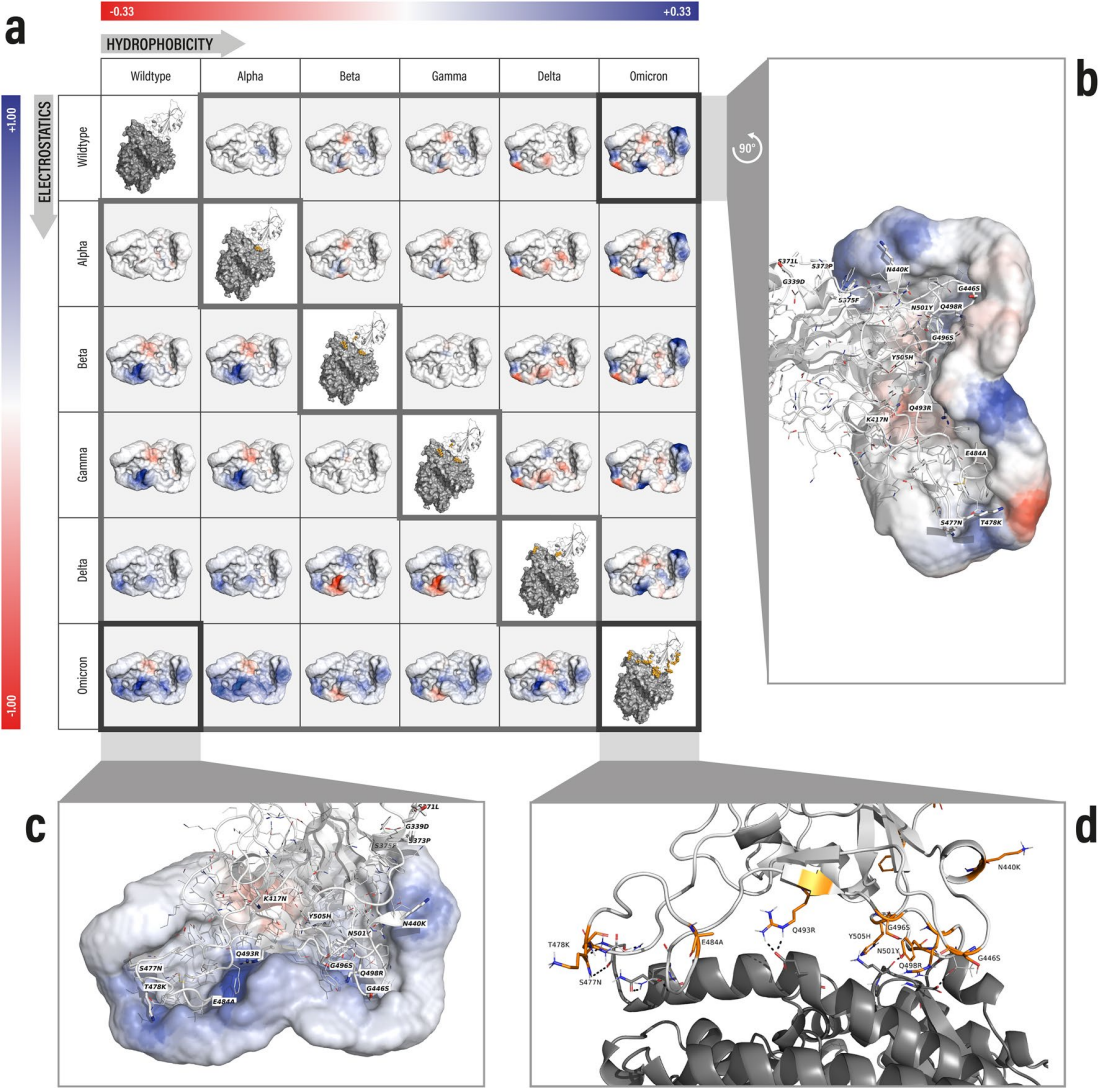
Surface representation of Halo difference point clouds aiming at pairwise spike VOC RBD comparisons. (**a**) Each difference Halo_*ij*_ was calculated by subtracting Halo field values of the variant associated with column *j* from Halo field values of the variant associated with row *i*. Upper triangle: hydrophobicity difference Halos, difference values are scaled and colored from red (− 0.33) to blue (+ 0.33), where white (= 0) corresponds to zero difference. Lower triangle: electrostatics Halo differences with values colored from red (− 1.0) to blue (+ 1.0). Diagonal: binding interfaces of spike RBD variants in complex with hACE2. (**b**) WT vs. Omicron hydrophobicity difference field. (**c**) WT vs. Omicron electrostatics difference field. (**d**) Omicron RBD-hACE binding interface revealing an additional H bond between spike R493 and hACE2 E35.

The influence of respective amino acid exchanges in the spike RBDs of the VOCs Alpha, Beta, Delta and Omicron regarding crucial properties can be visualized by Catalophore Halos. Halos are equidistant point clouds with values representing 16 physico-chemical property fields such as electrostatics, hydrophobicity, flexibility, potential energies, hydrogen-bonding acceptor/donor potentials, dissolvability, aromaticity, and others in a discretized manner, surrounding the molecular surface of the molecule that induces these fields^39^. Originally, they were developed for the calculation of fields inside buried protein cavities^40^. In the case of spike-RBD-hACE2 complexes, the Halo point clouds are calculated around the RBD surface at the hACE2 binding interface region and can be used for the comparison of spike variants, e.g., with regard to the effect of spike mutations on hACE2 binding. For that purpose, so-called difference Halo point clouds are generated from two aligned primary Halo fields (of two spike variants) by subtracting property values of one variant Halo from the values of the other. The concept and generation of Halo point clouds as well as difference Halos is explained in more detail in the methodology section and particularly in the supporting information with an illustrative Fig. S1. Figure 2a depicts pairwise differences in electrostatics (lower triangle) and hydrophobicity (upper triangle) of spike VOC RBD Halos. This representation makes it easy to recognize at a glance whether mutations have a noticeable influence on the RBD-hACE2 interface requiring comprehensive structural analysis.

In order to spot the most prominent differences between variants, we chose to present electrostatics and hydrophobicity Halo point clouds (out of a set of 16 calculated physico-chemical properties) represented as a difference Halo matrix in Fig. 2a. Each difference Halo_*ij*_ was calculated by subtracting Halo field values of the variant associated with column *j* from Halo field values of the variant associated with row *i*. Regarding electrostatics and hydrophobicity, the Omicron Halo, among all the other investigated variants, shows the most eye-catching differences compared to the wild type Halo (Fig. 2b,c). Interestingly, the Halos of Beta and Gamma appear to be more different from the wild type regarding electrostatics than Delta (Fig. 2a), whereas Alpha shows almost no difference regarding electrostatics. Regarding hydrophobicity, Alpha reveals a local increase of hydrophobicity (blue patch) in the region around its N501Y mutation compared to the wild type. Due to the recurrence of the same blue patch in the difference Halos of Beta, Gamma and Omicron compared to the wild type, it may be linked to alterations in biomolecular interactions and should be investigated in further detail. By visual comparisons of electrostatics and hydrophobicity difference Halos, Omicron seems to be akin to a combination of Beta and Delta with additional changes in the region where mutation N440K is located (Fig. 2b). The large Halo difference between Omicron and Delta is explained by mutations G339D, S371L, S373P, S375F, K417N, N440K, G446S, S477N, E484A, Q493R, G496S, Q498R, N501Y, Y505H that are only present in Omicron and the absence of L452R while only sharing T478K. At the binding interface, the new arginine replacing glutamine at position 493 in Omicron yields a new strong hydrogen bond with hACE2 amino acid E35 which is clearly reflected by the dark blue area (increase in positive charge) of the Omicron-WT difference Halo as illustrated by Fig. 2c. These depictions allow a rapid qualitative overview of the extent of physico-chemical changes at the RBD-hACE2 binding region. As a consequence, they allow a quick decision on which variants to prioritize for further resource-intensive structural and dynamics analysis, as for instance calculating binding affinities to hACE2 through the ESF we developed for this purpose.

### Development and optimization of a binding affinity model

We employed a two-parameter ESF closely related to the LIE method^41,42^ where the parameters are associated with van der Waals (VDW) and electrostatic interaction differences between the bound and unbound state. While different sets of physical assumptions lead to concrete values for these parameters in the original LIE method, they can also be used, based on a set of experimental data, to gauge the contribution of the corresponding terms to changes in Δ*G*^43–45^. Further details on the ESF setup as well as on various model assumptions and tests can be found in the methodology section below and in the supporting information.

In essence, our approach for a two-parameter model turned out to be a reasonable starting point as well as the most sensible model regarding the number of parameters. We confirmed this by a fit (for each case) to a suitable set of experimental data (as detailed below) as well as a round of leave-one-out cross-validation calculations that predict a set of Δ*G*. The correlation of this prediction with the experimental set is our concluding point of reference for model usefulness. As the analysis below shows, the resulting weight of the electrostatic contribution is very small and, within errors, consistent with zero. This may seem surprising, given the physical origin and motivation of this term, but straight-forwardly results from the fact that Coulombic energy difference values exhibit statistical errors of the same magnitude as the values themselves. The fitting procedure translates this weak signal-to-noise ratio into a very small weight for the electrostatic term.

The most obvious explanation for the large statistical width of electrostatic contributions is connected to the huge binding interface of the hACE2 ligand comprising around 35 amino acids (ca. 500 atoms), compared to simple protein-ligand interfaces with much smaller ligands and therefore much less noise. In addition, the Coulomb potential acts over longer ranges (compared to the Lennard–Jones potential which quantifies energy contributions due to short-range VDW forces) and its evaluation involves substantially more interacting atom pairs including many highly flexible surface side chains. The more rapidly declining VDW interactions appear to be more stable and significantly less sensitive to small displacements in atom positions^13^ and interface size. By design, the intermolecular Lennard–Jones potential is mostly affected by a narrow region of the protein-protein interface involving far fewer atoms than in case of long-range electrostatics. As a consequence, VDW contributions emerge as a clear signal and furthermore correlate well with experimental data. This observation indicates a strong dependence of the binding affinity on the steric fit of the two involved proteins. Thus, we are able to pioneer the application of an LIE related empirical scoring function to absolute protein-protein binding affinity prediction. Other published studies of LIE applied to protein-protein binding do exist but were limited to local subdomain analysis as for instance of regions around single mutations^46,47^.

Despite this good performance of a model with (effectively) a single parameter, we refrain from dropping the electrostatic term completely for the sake of generality. It is unclear, from our study alone, whether or not the term would be beneficial and/or significant in other cases.

**Figure 3:**
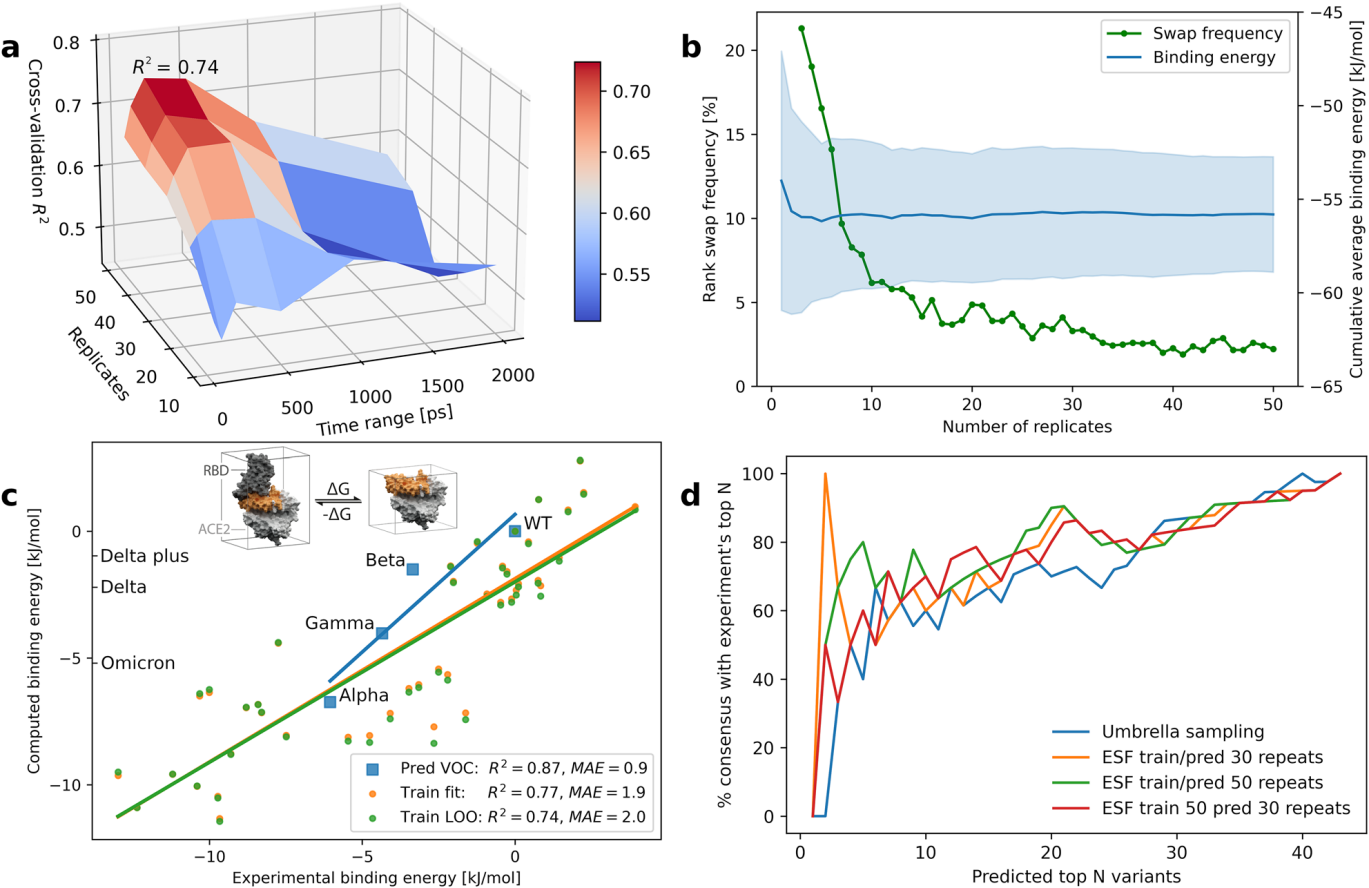
Empirical scoring function (ESF) development. (**a**) Optimization of run parameters, time range and number of replicates. (**b**) Binding energy convergence (rank-swap frequency and average cumulative energies with standard deviation range) vs. number of MD replicates. (**c**) Predicted (model fitting/cross-validation and VOC prediction) vs. experimental binding affinities of a 43 variants training set plus available VOCs calculated for hACE2 residues in a 5 Å vicinity of the spike RBD (brown colored region in embedded graphic). (**d**) Consensus fraction between predicted and experimental top N variants.

According to the last prediction column in Table S1 (visualized in Fig. 3a), the highest prediction accuracy and lowest error was achieved with 50 replicates of 200 ps molecular dynamics (MD) simulation trajectories, where, for model fitting and cross-validation respectively, *R*^2^ amounts to 0.77 and 0.74 with an average error of less than 1.9 and 2.0 kJ/mol.

A corresponding scatter plot of fitted and cross-validated vs. experimental binding energies ΔΔ*G* (relative to the wild type WT) is depicted in Fig. 3c. Fortunately, the experimental ranking of our training set variants is very well reflected by these model predictions. In addition, the distribution of experimental Δ*G* values in terms of mean and standard deviation (− 55.76 ± 4.7 kJ/mol) agrees extraordinarily well with those of the fitting and cross-validation procedure, both amounting to − 55.8 ± 3.9 kJ/mol. Furthermore, overfitting can safely be considered as minimal since corresponding points (variants) of fitting and cross-validation plots as well as both regression lines are lying very nearby. The results in Fig. 3a have been obtained with the optimal fitted weights, *w*^vdw^ = 0.76472 and *w*^elec^ = 0.02355, associated with model A.1. Having applied this predictive model to an unpublished internal set of 21 hACE2 variants in complex with the wild type for which EC50 values were available, we obtained a remarkable prediction accuracy of *R*^2^ = 0.61. Prior to that, EC50 values had been converted to binding free energies in analogy to *K*_D_ values.

Considering the number of replicates needed for a sufficient convergence of the energies and consequently of affinity-based variant rankings, the plot in Fig. 3b indicates moderate stability already from 30 replicates on, although MAE and *R*^2^ are still slightly worse compared to 50 replicates (Tables S2–S4). We can safely assume a continuous growth of the model accuracy with additional replicates, at least for the ESF group A.x (as defined in Table S1). However, with regard to the required run time (currently 1.1 days on a Amazon AWS C6i.16xlarge instance in case of 200 ps trajectories), 50 replicates seem to be a reasonable choice if we put more emphasis on accuracy. For this convergence analysis we determined the fraction of variant pairs associated with a swap in their accumulative energy order while increasing the number of replicates. All frequencies in percent were obtained via division by the total number of variant pairs *n* = 43^2^ = 1849. In more urgent cases, 30 replicates would take around 15 h to run at a slightly lower accuracy.

Another major quality aspect of binding affinity models is related to the predicted ranking of VOCs, as we strive for high consensus between predicted and experimental top-N variants. Figure 3d illustrates the fraction of variants among the top N predicted ones that are also included in the top N experimental variants of the training set. We compared the performance of an empirical model (a) trained with 50 replicates and applied to predictions on the basis of 50 replicates as well, (b) same as (a) but using 30 replicates for both steps, and (c) a mix of (a) and (b), namely model training with 50 but predictions on 30 replicates. Finally, (d) a non-empirical prediction model published by Singh et al.^13^ in 2020 and based on umbrella sampling along with weighted-histogram analysis was applied to the same training set of 43 variants. As expected, the highest consensus, especially in the range of up to top 5, was obtained by models trained with 50 replicates, namely cases (a) and (c) corresponding to the green and red plots (where green is sometimes hidden behind the red line). Regarding predictions of training set items, up to 80–100% of the top 3–5 are in agreement with experimental top 3–5 candidates according to the ESF approaches (a) and (b) and significantly less consensus along the entire range of N variants using the umbrella sampling method, in particular less than 50% among the top 5 variants.

### Affinity model application to VOCs

Using our final predictive model calibrated with the optimal weights,

$$\Delta G = 0.76472\Delta E^{vdw} + 0.02355\Delta E^{elec} ,$$

we estimated binding affinities for VOCs that had emerged during the past year: Alpha, Beta, Gamma, Delta, and most recently Omicron. However, we have to bear in mind that our structural variant models only comprise the spike RBD rather than the entire spike trimer. Table 1 compares our calculated relative binding energies ΔΔ*G* along with *K*_D_ ratios (both in relation to the wild type) derived at 310 K temperature with experimental values for WT, Alpha, Beta, and Gamma recently determined by Barton et al. through surface plasmon resonance^32^.

**Table 1:** SARS-CoV-2 VOCs relative binding free energies and ratios of dissociation constants in relation to the wild type predicted by our empirical scoring function compared to results determined through surface plasmon resonance by Barton et al*.*^32^ for a subset of these VOCs.

Variant	Experimental affinities			Predicted	
	*K*_D_ [nM]	*K*_D_ ratio	ΔΔ*G* [kJ/mol]	*K*_D_ ratio	ΔΔ*G* [kJ/mol]
WT	74.4	1	0	1	0
Alpha	7.0	0.09	− 6.1	0.07	− 6.7
Beta	20.0	0.27	− 3.4	0.56	− 1.5
Gamma	13.5	0.18	− 4.4	0.21	− 4.0
Delta	*n.d.*			0.69	− 0.9
Delta plus	*n.d.*			0.42	− 2.2
Omicron	*n.d.*			0.13	− 5.2

According to the results in Table 1 and Fig. 3c, the predicted order of dissociation constants for WT, Alpha, Beta and Gamma exactly reflects experimental findings^32,48^ and yields a coefficient of determination *R*^2^ of 0.88 based on ΔΔ*G* values. In particular, with a 14-fold decrease compared to the wild type dissociation constant, the most outstanding binding affinity among all VOCs was predicted for Alpha. This is also in line with lab data^32,48^ revealing around tenfold binding affinity increase and reports^49^ stating, for the UK between October and November 2020, a 70–80% rise in transmissibility with respect to the wild type reference.

For the new SARS-CoV-2 Omicron spike variant, our studies reveal a similarly remarkable 7.7-fold increase of binding affinity compared to the wild type. In terms of binding strength it therefore ranges between Alpha and Gamma. A significantly lower binding affinity, somewhere around Beta but lower than the wild type, was predicted for Delta and Delta plus. Moreover, Delta plus (having K417N in addition to the amino acid exchanges found in Delta) was predicted to have a lower affinity than Delta, which is supported by experimental results revealing weaker binding due to the presence of K417N^48,50^. In addition, the authors allude to an increase of immune evasion caused by this mutation. It should be noted that Omicron as well includes this mutation possibly explaining its lower binding affinity compared to Alpha and thereby indicating a strong tendency to immune evasion for Omicron. The relative order of WT, Delta and Omicron is also in line with Docking results recently published by Kumar et al.^51^, although energy magnitudes and therefore differences given in that article translate to a *K*_D_ ratio of 10 million for Omicron compared to the wild type.

## Discussion

Following the trade-off model of virulence^52^, the SARS-CoV-2 virus, like other viruses^53^, is constantly evolving^54^ by modulating the rate of infectious transmission, higher virulence, and higher virus production in order to improve viral fitness. In this context, viral immune evasion is an evolutionary strategy to allow for the coexistence of viruses and their hosts^55^ as shown with artificial polymutant SARS-CoV-2 spike protein pseudotypes that resisted antibody neutralization to a similar degree as circulating VOCs^56^.

Typically, viruses adapt to a specific host. However, when hosts fluctuate in time or space, generalist viruses may evolve as well^57^. It is hypothesized that during this process, intermediate virulence maximizes pathogen fitness as a result of a trade-off between virulence and transmission^58^. Although it is often speculated that virulence of viruses will finally decrease over time, the pandemics of our past, such as SARS in 2003 and flu in 1918–1920, 1957, 1968 and 2009, decayed due to other reasons and not because the viruses evolved to cause milder symptoms^59^.

During the ongoing COVID-19 pandemic it is therefore required to continuously adapt mitigation strategies, drugs, and vaccines in order to mitigate the impact during an early phase of the establishment of this virus. The world has employed massive sequencing programs (based on infected patients, animal, or large-scale sewage monitoring samples) that continuously provide information on changes in the viral genome. While this ensures an early detection of altered genomes, it by principle lacks information about the changed characteristics of the emerging variants. Therefore, only further and detailed investigations like structural analysis and the prediction of the biological impact of occurring mutations allow us to be one step ahead of the virus by helping to forecast altered virulence, transmissibility, or immune evasion potential.

Given the enormous number of emerging genomes, a fast tool such as Catalophore Halos can help to rapidly identify changes in RBD/hACE2 interface fields and guide a decision of which variants to simulate in full atomistic detail. Difference Halos, in particular, provide qualitative information about the changes of specific physico-chemical properties when comparing surfaces of SARS-CoV-2 VOC RBDs. Changes in electrostatics or hydrophobicity, as observed for the Omicron variant (Fig. 2), may be associated with significant alterations in biomolecular interactions and the viral pathogenicity. This can affect viral transmissibility, the binding affinity between RBD and hACE2, immune escape or the range of hosts. Omicron difference Halos indicate a significant shift to positive electrostatic charges at the Omicron RBD surface compared to the other VOCs, which is in line with previous reports^60,61^. Specifically, T478K, Q493R, Q498R, N440K and E484A cause positively charged patches or decrease negative charges at the RBD surface, which is clearly visible in the difference Halos. R493 might interact with negative charges around E35, whereas R498 might be attracted by D38 of hACE2. Further, K417N, occurring in Omicron and Beta RBD, was shown to disrupt the salt bridge to D30 of hACE2 and to neutralizing antibodies, which has also been described for K417T of Gamma. Thus, these mutations could facilitate the immune evasion of these virus variants but weaken the binding to hACE2^50,62^. N501Y was described to be a key residue in hACE2 binding as it favors a hydrophobic cation-π interaction to K353 of hACE2 and a π-π stacking interaction with hACE2 Y41^62^. The local increase of hydrophobicity caused by N501Y is clearly visible in the Alpha, Beta, Gamma and Omicron difference Halos (difference to the wildtype, Fig. 2a). We further examined the difference Halo outcome by focussing on the effects on overall RBD-hACE2 binding affinity.

Based on the LIE method, we have developed an empirical binding affinity estimator that predicts pretty accurate binding energies for spike-RBD-hACE2 complexes at moderate wall-clock run times, especially using massive cloud computing facilities. With the necessary caution, this technique helps to raise flags to indicate potential higher infectiousness. The precision of our SARS-CoV-2 spike-RBD-hACE2 binding affinity model is mainly due to the high number of replicates used to achieve a satisfactory convergence in binding energies and both a solid and sufficiently large training set from one source, thanks to Zahradnik et al.^34^ With a mean absolute error of less than 2 kJ/mol, our predictions are in the same error range as experimental binding energy methods^63^. To the best of our knowledge, this is the first application of an LIE-based model to the prediction of the binding free energy of an entire protein-protein interface and it is interesting to observe a very uncommon relation between VDW and electrostatic weights. The extremely low binding energy contribution of the latter, already obtained in other published fitting approaches^64^, is obviously caused by the enormous background noise in the case of the huge interface consisting of roughly 500 atoms and a distinct sensitivity of the Coulomb potential to positional displacements causing large fluctuations as published in the supplementary information of a recent article^13^. This uncertainty cannot be compensated by using 50 trajectory replicates and becomes even worse with increasing interface cutoffs or by handling the spike RBD as the ligand. The latter effect is most likely due to the exclusive presence of loops rather than defined secondary structure elements and the absence of hACE2 as a structure-stabilizing binding partner. Fortunately, force field based VDW interaction energies of the “small” 5 Å interface region reveal remarkable stability over the 50 replicates and, on average, correlate well with experimental binding affinities. A reasonable explanation for this stability and correlation is the mathematical short-range nature of the Lennard-Jones potential and, thus, a strong dependence of the binding affinity on the proteins’ steric fit. Nevertheless, here as well, the model accuracy strongly suffers under background noise if the interface is specified significantly larger.

The binding affinity is usually quantified by the equilibrium dissociation constant (*K*_D_), which is used to evaluate and classify the strength of biomolecular interactions. The smaller the *K*_D_ value, the greater the binding affinity of the ligand for its target. The actual *K*_D_ value relevant to a concrete biological situation depends on the physiological environment, e.g., salt concentration, temperature, or pH. Therefore, both the measured and modeled absolute *K*_D_ values are only valid within the observation range, making the comparison between unrelated data sources complex. Thus, we reported *K*_D_ ratios relative to the wild type which clearly show a structural, biologically explainable increase in the binding affinity of all VOCs, particularly pronounced for Alpha and the new VOC Omicron.

Most of our computational results associated with model training as well as application to SARS-CoV-2 VOCs are in agreement with experimental findings. For instance, the relative order of binding affinity predictions for spike VOCs are exactly in line with experimental observations^32,48,50^ of WT, Alpha, Beta, and Gamma. Moreover, the outstanding binding strength associated with Alpha (N501Y) as well as the decrease in binding affinity due to the immune escape mutation (K417N/K417T) in addition to N501Y in case of Beta and Gamma is also perfectly reflected. According to our findings, Omicron achieved a substantially higher increase in binding affinity than all other VOCs except for Alpha. Compared to Beta and Gamma, Omicron bears 13 other mutations, six of which were described to enhance binding to hACE2^13,35–38,60^. Other mutations within the Omicron RBD were proposed to weaken the binding affinity to hACE2 and further enhance immune escape due to weaker binding to antibodies^60^. This could explain the second strongest binding affinity to hACE2 for Omicron next to Alpha, despite the drastic changes in electrostatics and hydrophobicity observed in the Omicron difference Halos (Fig. 2a). Since the initial submission of this publication in early December 2021, additional experimental binding affinities for Omicron have been published by different groups. In Table S5 we took the opportunity to compare some of these experimental Omicron RBD-ACE2 binding data to our predictions. In contrast to Han et al., who described the Omicron binding affinity to be lower compared to the wild type^60^, other recent publications showed an RBD binding affinity to ACE2 approximately two times higher compared to the wild type^65–67^ and K_D_ values (VOC RBD to hACE2) from Cameroni et al*.*^67^ correlated well with our predictions. Our initial conclusion still remains true, this new variant requires our special attention and monitoring.

In summary, our ESF derived from the LIE model allows us to estimate high-level binding affinities between the spike RBD and hACE2 regions. However, although we found the model fairly accurate when compared to experimental values, predicted affinities should be interpreted as binding ”trends” instead of absolute *K*_D_ values. As noted elsewhere^16–18^, increased binding affinity often leads to increased infectivity of SARS-CoV-2. Given the experience with the global wave of VOC Delta, it is therefore now necessary to closely monitor the spread and impact of Omicron in the coming months. Initial medical reports^68^ from South Africa^69^, Botswana, and Europe^70^ with a relatively small, non-representative sample of patients revealed a changed clinical picture with a comparatively mild course of the disease^71^. As population immunity increases, either through infection or vaccination, steady modulation of immune-evading mutations might contribute to a permanent establishment of SARS-CoV-2, wherein Omicron apparently shows the potential of playing a significant role.

## Methodology

### Analysis of spike RBD diversity

For the analysis of RBD diversity, all spike protein sequences that were available by December 6th 2021 at GISAID^21^ were downloaded in FASTA format. Processing and analysis of the sequences was performed employing in-house tools in Python. The spike RBD sequences (residues 319–541) were extracted by splitting each spike protein sequence after the amino acid motif “TSNF” and before “NFNG”, which refer to the RBD flanking regions. Only sequences consisting of 223 residues (the length of the RBD) were accepted, i.e., insertions and deletions were not considered. Each residue of the retrieved RBD sequences was compared with the respective residue in the reference RBD^21^. Every mismatch except for low-quality or sequencing error residues (indicated by “X”) was counted as one mutation. For the analysis of spike RBM diversity, residues 437–508 were considered.

### Preparation of spike RBD-hACE2 structures

A 2.47-Å-resolution crystal structure of the SARS-CoV-2 spike RBD bound to hACE2 (PDB-Code: 6m0J) was used as a starting structure. Employing the molecular modeling and MD simulation package Yasara^72^, conformational stress was removed by steepest descent energy minimization followed by simulated annealing (timestep 2 fs, atom velocities scaled down by 0.9 every 10th step). For this purpose, we selected the AMBER14 force field^73^ applying an 8 Å force cutoff. SARS-CoV-2 spike-RBD variant sequences were constructed based on the SARS-CoV-2 spike RBD starting sequence from the wild type lineage. Mutant structures were built using homology modeling by implementing Yasara with a maximum of five alignment variations per template and not more than 50 conformations tried per loop. The minimized starting structure served as a template. The final input files contained residues 333–526 of the respective SARS-CoV-2 RBD and residues 19–615 of hACE2 coordinating one zinc ion.

### Molecular dynamics simulations

Again using Yasara, each spike RBD-hACE2 complex was centered in a cuboid simulation box under periodic boundary conditions with a solute-wall distance of 5 Å on every side. This box was filled with explicit solvent molecules of the TIP3P model^74^, approximating 0.997 g/mol water density and with 0.9% sodium chloride ions as well as additional ions for system neutralization. Protein structure topologies and energetics were parameterized according to the Amber14 force field. The system pressure was set to 1 bar using the Manometer1D setting of Yasara, while temperature coupling to 310 K was achieved by velocity rescaling as described by Krieger et al.^75^ Long-range electrostatic interactions were treated through Particle Mesh Ewald^76^ summation (PME) with an 8 Å cutoff. Our protocol consisted of a steepest descent energy and successive simulated annealing minimization step (following Yasara’s standard minimization protocol) and a final production run of 200 ps (respectively 2 ns in case of model optimization) per replicate using a step size of 1 fs for intramolecular and 2 fs for intermolecular forces. Afterwards we extracted intermolecular interaction energy contributions, *E*^vdw^ and *E*^elec^, caused by van der Waals and electrostatic forces, respectively. In addition, hACE2 in its dissociated state underwent the same procedure from simulation box generation up to production MD in order to have both bound and unbound states available for the development of a predictive model.

### Development of a binding affinity model

From the various MD based approaches to binding affinity estimation, the empirical linear interaction energy (LIE) method developed in the 90s by Åqvist et al.^41,42^ has provided a remarkable trade-off between predictive accuracy and computational effort^44,63,77,78^. In contrast to complex methods mimicking thermodynamic reaction paths represented by high degrees of decomposition and numbers of long trajectories, it exclusively relies on short simulations of a bound/associated (PL) and an unbound/dissociated state (L) of the ligand as depicted by the embedded graphic in the upper left corner of Fig. 3a. Starting from the linear response approximation^79^, the developers pointed out a strong relationship between the Gibbs free energies of binding on the one side and, on the other side, average (denoted by angle brackets ⟨⟩) differences Δ*E*^vdw^ and Δ*E*^elec^ of the ligand's interaction energies with its surrounding atoms, namely protein and solvent atoms in the bound and solely solvent atoms in the unbound case.

$$\Delta G = \frac{1}{2}\left[ {\left\langle {E^{elec} } \right\rangle_{PL} - \left\langle {E^{elec} } \right\rangle_{L} } \right] + \alpha \left[ {\left\langle {E^{vdw} } \right\rangle_{PL} - \left\langle {E^{vdw} } \right\rangle_{L} } \right].$$

The coefficient alpha was calibrated with respect to a small training set of protein-ligand complexes. Subsequent LIE studies revealed significantly better correlations when both coefficients, α and β, were treated as empirical parameters, possibly extended by further features, and fitted to available training sets with known binding affinities^43–45^

$$\Delta G = \beta \left[ {\left\langle {E^{elec} } \right\rangle_{PL} - \left\langle {E^{elec} } \right\rangle_{L} } \right] + \alpha \left[ {\left\langle {E^{vdw} } \right\rangle_{PL} - \left\langle {E^{vdw} } \right\rangle_{L} } \right].$$

Our final ESF of the spike-hACE2 binding indeed makes use of two empirical parameter weights *w*^vdw^ and *w*^elec^ (used instead of α and β and having omitted the angle brackets for the sake of clarity):

1 $$\Delta G = w^{vdw} \;\Delta E^{vdw} + w^{elec} \;\Delta E^{elec} .$$

Since we are dealing with two interacting proteins rather than a protein-ligand system, we tested both proteins independently as ligand molecules simulated in complex as well as freely in solvent. It must also be noted that due to the huge size of these “ligands” and in order to reduce the impact of noise, only interactions of ligand amino acids with at least one atom within a 5 Å (and 10 Å for comparison) environment of the binding partner (e.g. brown colored area of hACE2 object in embedded graphic of Fig. 3a) were evaluated upon energy computation in the bound as well as unbound case.

As discussed in the Results and Discussion sections, treating hACE2 as a ligand significantly increased the accuracy of our model, although mutational changes are associated with RBD, whereas the hACE2 sequence remains constant within the training set. Moreover, our model (Eq. 1) treats both energy contributions as empirical parameters fitted to a training set where, in particular, the optimal electrostatic weight deviates enormously from its original value close to 0.5. Since our affinity model deviates from the LIE protocol in several aspects, we decided to call it an empirical scoring function rather than an LIE model, which is nevertheless closely related to it.

For model development and evaluation we employed a sufficiently large training set of 43 spike RBD variants including the wild type (PDB ID 6M0J) in complex with hACE2 for which Zahradnik et al. had recently published *K*_D_ values obtained through yeast surface display titration^34^. The number of mutations per variant ranges from one to seven, with a maximum distance to hACE2 amounting to 2.5 nm. Using a linear regression model 

along with least-squares fitting

$$x = \left( {A^{T} A} \right)^{ - 1} A^{T} y$$

interaction energy weights of the empirical model were fitted to experimental Δ*G*^exp^ values derived from *K*_D_ values at a temperature of *T* = 310 K and using the gas constant *R*:

2 $$\Delta G = RT\ln K_{D}$$

Gibbs free energies of binding for new variants were then predicted by summing up the two weighted interaction energy contributions according to Eq. (1) and translated back to *K*_D_ values by inverting Eq. (2).

Our predictive model was validated and optimized through leave-one-out cross-validation, ensuring that none of the 43 variants was inside the training set used to predict its binding energy. Mean absolute errors and squared Pearson correlation coefficients served as measures for model accuracy. In order to achieve a satisfactory binding energy convergence and increase model accuracy, we produced and analyzed 50 replicates of 2 ns MD trajectories and averaged the two interaction energy terms.

### Catalophore Halo analysis

Spike RBD Halos were calculated as fields of physico-chemical properties, represented by 3D point clouds covering the entire outer side of the molecular surface of each RBD variant in complex with hACE2. Molecular surfaces were hereby defined with a probe radius of 1.4 Å around the RBD atoms’ VDW radii, and the point clouds on the RBD surface had a thickness of 5 Å. These full size Halos were cropped down to the binding interface region, that is the space which is occupied by atoms of the binding partner hACE2 plus corresponding VDW radius plus an additional 5 Å extension around these atoms. It is important to note that hACE2 is not influencing the properties, hence solely the shape, of the spike RBD Halos. The grid spacing of the point clouds was set to 0.75 Å.

Halo point clouds of all spike RBD variants were aligned in space using the iterative closest point algorithm and by taking into account both coordinates and property values. Afterwards, difference Halo point clouds were generated for each pair of two RBD variant Halos under investigation by placing a new point at the geometric center of each pair of two corresponding points of the two primary Halo point clouds. For the definition of pairs of corresponding points we used a distance cutoff of 0.5625 Å. Finally, property values for the new difference cloud points were calculated as the difference of the values associated with the corresponding pair of two primary points, thereby subtracting values of the points in the first primary point cloud from the values of the second primary point cloud. The concept of Catalophore Halos is described in more detail in the supplementary information.

### Computational details

The simulation performance was optimized in cooperation with Amazon Web Services (AWS), who supplied the necessary cloud infrastructure in the framework of the diagnostic development initiative. We used clusters of AWS Elastic Computing (EC2) × 86 instances of the C5 und C6i families running 64-bit Amazon Linux 2 with AMI Kernel 5.10, e.g. c6i.8xlarge (32 virtual CPUs), c5.4xlarge (16 vCPUs) and c6i.16xlarge (64 vCPUs) equipped with Intel Xeon Scalable processors. With this configuration the simulation of each variant was completed within 3 to 6 days. Initial proof-of-principle simulations were executed with GROMACS 2021.2 on ARM-based AWS Graviton instances c6g.8xlarge (32 vCPUs).

## Supplementary Information

Supplementary Information.

## Data Availability

Publicly available datasets were analyzed in this study. This data can be found here: https://www.gisaid.org/. Input and final structure files as well as Pandas Dataframes of interaction energies exported as Python Pickle files generated within this work are available for download at 10.6084/m9.figshare.17129771.
