# Supplementary material for: Structural bioinformatics analysis of SARS-CoV-2 variants reveals higher hACE2 receptor binding affinity for Omicron B.1.1.529 spike RBD compared to wild type reference
Source: Sci Rep. 2022 Aug 25;12:14534. doi: 10.1038/s41598-022-18507-y (PMC9406262; doi:10.1038/s41598-022-18507-y)
Supplement: Supplementary file 1 — Supplementary Information. [file 41598_2022_18507_MOESM1_ESM.docx]

Supporting information

**Structural-bioinformatics analysis of SARS-CoV-2 variants reveals higher hACE2 receptor binding affinity for Omicron B.1.1.529 spike RBD compared to wild type reference.**

Vedat Durmaz^1‡*^, Katharina Köchl^1‡*^, Andreas Krassnigg^1^, Lena Parigger^1^, Michael Hetmann^2,3^, Amit Singh^1,2^, Daniel Nutz^1^, Alexander Korsunsky^1^, Ursula Kahler^1^, Centina König^1^, Lee Chang^4^, Marius Krebs^5^, Riccardo Bassetto^5^, Tea Pavkov-Keller^2^, Verena Resch^1^, Karl Gruber^2,6^,
Georg Steinkellner^1,2*^ & Christian C. Gruber^1,2*^

^1^Innophore GmbH, 8010 Graz, Austria

^2^Institute of Molecular Biosciences, University of Graz, 8010 Graz, Austria

^3^Austrian Centre of Industrial Biotechnology, 8010 Graz, Austria

^4^AWS Diagnostic Development Initiative - Global Social Impact, 98109 Seattle, WA USA

^5^Amazon Web Services EMEA SARL, 80807 Muenchen, Germany

^6^Field of Excellence BioHealth – University of Graz, 8010 Graz, Austria

^‡^Both authors contributed equally to this manuscript.

*Correspondence should be addressed to ([christian.gruber@innophore.com](mailto:christian.gruber@innophore.com) or [georg.steinkellner@innophore.com](mailto:georg.steinkellner@innophore.com))

**Catalophore Halo technology**

With Catalophore Halos we refer to contiguous equidistant point clouds associated with value sets representing 16 physico-chemical property fields (including electrostatics, hydrophobicity, flexibility, potential energies, hydrogen-bonding acceptor/donor potentials, dissolvability, aromaticity and others) in a discretized manner[^1^](https://www.zotero.org/google-docs/?F31XDC). They are located on the outer molecular surface of the protein that induces these fields. Similar proteins such as mutational variants most likely induce slightly different property fields in terms of both point cloud shape and corresponding values at the single points. Halo point clouds are able to identify physico-chemical characteristics such as charged surface spots or helical dipole moments[^2–4^](https://www.zotero.org/google-docs/?hO7YGa) that are hardly captured by sequence or structure patterns.

The foundation for the Halo approach lies in the Ligsite algorithm[^5^](https://www.zotero.org/google-docs/?Pwp1Ev) used for the identification of buried protein cavities and was extended to calculate properties within these enclosed spaces. Compared to those Catalophore cavities[^6^](https://www.zotero.org/google-docs/?FXdO6K) mainly used for enzyme discovery and drug design[^7^](https://www.zotero.org/google-docs/?dxPOED), Catalophore Halos are constructed on the entire outer protein surface as a layer of typically 5 Å thickness. For known protein-protein complexes, Halos can then be trimmed to cover only the most relevant area under investigation, for instance the hACE2 binding interface region of the spike RBD as shown in Figures 2b and 2c of the main article. This was achieved by cropping all points that were located outside the molecular surface of the binding partner hACE2. More precisely, Halo points farther away from their respective next hACE2 atom than the VDW radius plus an additional 5 Å were omitted. However, the values of the Halo scalar field are only induced by spike RBD atom properties. It is important to note that, on purpose, the counterpart protein (hACE2) is not influencing the properties of the spike RBD Halo.

Since the shape and value distribution of Halo fields vary between mutational variants, Halos can be used for the comparison of spike variants and thus the effect of spike mutations with respect to hACE2 binding. Indeed, Halo point clouds become especially powerful for the comparison of property distributions at the binding interface of similar molecules (spike RBD variants) that are likely to form a complex with the same target (hACE2). Thus, so-called “difference Halos” particularly highlight regions of locally differing physico-chemical property distributions when comparing two selected protein variant Halos. Difference Halos are generated from two primary Halo fields, as illustrated in the upper part of Figure S1 for SARS-CoV2 wild type and Omicron, by subtracting property values of one variant Halo from the values of the other, resulting in the difference Halo at the bottom of Figure S1. Prior to difference calculation, both point clouds are aligned in space using the iterative closest point algorithm thereby taking into account atomic coordinates as well as property values. More precisely, difference Halos are generated through linear interpolation as follows:

1. Creation of a correspondence list of point pairs of the two underlying clouds taking into account only pairs of points with a distance less than a cutoff value (default 0.5625 Å) in order to avoid boundary artifacts
2. Generation of a new difference point at the geometric center of each pair of two corresponding points
3. Assigning property values to each new difference point calculated as the difference of the two corresponding primary point values (Figure S1)

It should be noted that due to the cutoff in step 1), difference point clouds only cover a tight overlap region of the two primary proteins thereby neglecting regions of differing shapes. Without this security measure, the algorithm would produce unreasonable values in non-overlapping regions far away from the actual primary points interpolated at these regions. At least in the case of differing shapes far away from the surface, this issue may be tackled by increasing the thickness/size of primary Halo point clouds at the cost of computational time. However, differing Halo shapes right at the surface can hardly be compensated by this procedure since the Halo fields, mostly calculated through atomistic force fields, would yield extremely unphysical values at points under the surface (too close to the atoms of the inducing protein).


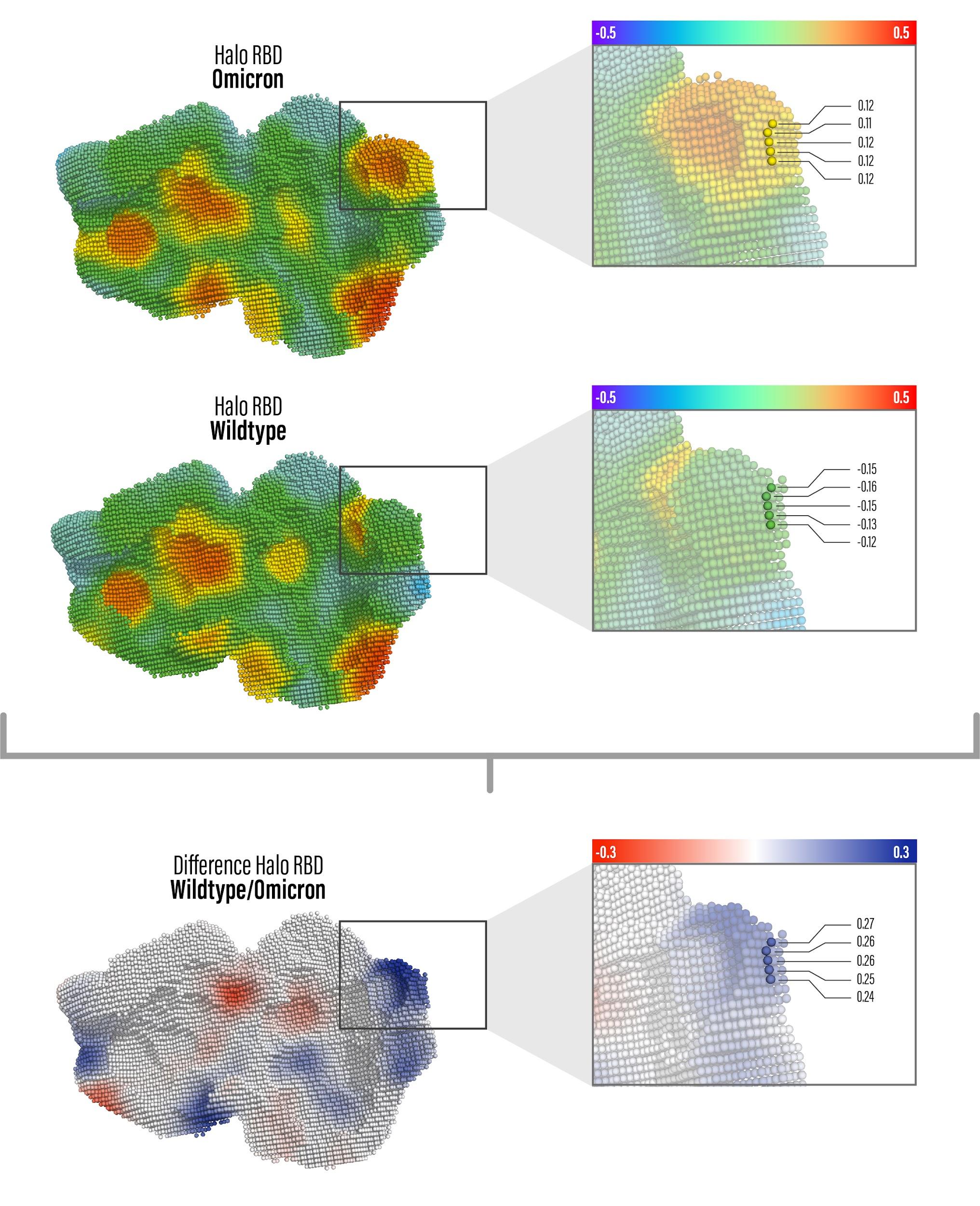


**Figure S1.** Illustration of difference Halo (bottom) construction from hydrophobicity Halo point clouds of two SARS-CoV-2 variant RBDs, wildtype and Omicron, as an example. Each difference point was determined as the geometric center of the two corresponding primary points. Its hydrophobicity difference value was calculated through linear interpolation by subtracting the value of point (cloud) 2 from point (cloud) 1.

**Evaluation of empirical binding energy models**

| **Model** | | **fitted weights** | | | ***R*^2^** | | **MAE** | | **convergence** |
| --- | --- | --- | --- | --- | --- | --- | --- | --- | --- |
| **Properties** | **name** | **coul** | **vdw** | **constant** | **fit** | **loo** | **fit** | **loo** |  |
| ACE2 ligand and  5 Å interface | A.1 (200 ps) | 0.024 | 0.765 |  | 0.768 | 0.742 | 1.87 | 1.96 | yes |
|  | A.2 | 0.045 | 1.079 | 24.4 | 0.790 | 0.760 | 1.71 | 1.84 | yes |
|  | A.3 |  | 0.807 |  | 0.743 | 0.729 | 1.89 | 1.94 | yes |
|  | A.4 |  | 0.895 | 6.537 | 0.743 | 0.721 | 1.91 | 2.00 | yes |
| ACE2 ligand and  10 Å interface | B.1 | -0.003 | 0.737 |  | 0.670 | 0.633 | 2.28 | 2.40 | no |
|  | B.2 | -0.013 | 0.565 | -14.5 | 0.681 | 0.624 | 2.24 | 2.42 | no |
|  | B.3 |  | 0.732 |  | 0.665 | 0.649 | 2.30 | 2.36 | no |
|  | B.4 |  | 0.709 | -1.8 | 0.665 | 0.627 | 2.30 | 2.43 | no |
| RBD ligand and  5 Å interface | C.1 | 0.050 | 0.713 |  | 0.554 | 0.525 | 2.55 | 2.66 | no |
|  | C.2 | 0.042 | -0.234 | -69.9 | 0.681 | 0.639 | 2.07 | 2.22 | no |
|  | C.3 (50 ps) |  | 0.740 |  | 0.373 | 0.407 | 5.63 | 5.77 | no |
|  | C.4 (50 ps) |  | -0.837 | -118.9 | 0.410 | 0.359 | 2.86 | 2.99 | no |
| Original LIE | D | 0.43 | 0.18 |  | 0.113 |  | 12.3 |  | no |

**Table S1**. Weight coefficients, squared correlation coefficients (*R*²) and mean absolute errors (MAE) of least squares fitting (fit) and leave-one-out (loo) cross-validation for various empirical binding energy models. Each model outcome was averaged over 50 replicates of 500 ps MD trajectories apart from Model C energy equations without electrostatic which yielded highest accuracies with 50 ps MD runs, and Model A with electrostatic and VDW parameters performing best at 200 ps. A monotonic accuracy increase with the number of replicates is indicated by the convergence column.

Upon model development, we tested various combinations of three parameters corresponding to electrostatic and van der Waals forces (VDW) as well as a constant energy term which are typically used in LIE models[^8,9^](https://www.zotero.org/google-docs/?pqz2UG) resulting in a set of different models as shown in Table S1. In order to substantiate our optimal empirical scoring function (ESF) incorporating the two force field based parameters, electrostatics (coul) and VDW (vdw), we compared all models with respect to leave-one-out cross-validation (LOOCV) of the training set and prediction of a test set. In addition to the purely empirical treatment, we fixed the electrostatics and VDW coefficients directly to standard LIE values from the literature, which produced the model with the poorest accuracy compared to all others with fitted coefficients as shown in supplementary Table S1 (model D). In contrast, we obtained high correlations with experimental binding energies by using a combination of two parameters associated with VDW and electrostatic interactions.

Moreover, according to Table S1, the best-correlating models (A.1-A.4) were obtained when treating hACE2 as the ligand in an unbound simulation and keeping its corresponding binding interface small. To be more precise, the results obtained with models A.x by specifying, 1), only those amino acids as part of the interface of which at least one atom was lying within a 5 Å vicinity, and 2), of the spike RBD (synonymous with treating ACE2 as the ligand) agree substantially more with experimental results than those obtained with a 10 Å cutoff (models B.x) or with the RBD treated as ligand (models C.x). In addition, as indicated by the convergence column in Table S1, model group A.x reveals a clear monotonic increase in the accuracy with increasing replicate numbers, which is a good indicator of numerical stability, whereas models of type B.x and in particular of type C.x do not.

The most obvious explanation is the statistical width of the energy distributions which is considerably larger in case of an interface consisting of more than 80 amino acids (10 Å cutoff) compared to an 33 amino acid interface (5 Å cutoff). With this optimal model the coefficients of determination *R*^2^ amount to values between 0.77 and 0.79 for fitting (fit) and between 0.74 and 0.76 for cross-validation (loo) depending on whether a constant parameter was included (model A.2 with slightly larger values) or not (A.1). The mean absolute error (MAE) is below 2 kJ/mol in any of these cases of model group A, which is impressive too. Interestingly but probably not surprisingly[^10^](https://www.zotero.org/google-docs/?XNWeaf), most of the variance in experimental binding energies is sufficiently explained by VDW energy differences as indicated by *R*^2^ fit/loo of 0.743/0.729 for that one-parameter model A.3, which is only slightly less than what the model including additional electrostatic contributions yields (A.1 with *R*^2^ fit/loo of 0.768/0.742). Including a constant in addition to both physical parameters further increases the correlation to a maximum (A.2 *R*^2^ fit/loo of 0.79/0.76). However, for two particular reasons we decided to work with the parameter setup of model A.2 (VDW and electrostatics): first, the constant reveals high instability as it fluctuates between -25 and 25 kJ/mol upon different combinations of time ranges and numbers of replicates and, second, the prediction of VOC binding affinities and especially their ranking was best reproduced by model A.1. Also, its optimal predictive accuracy results from 200 rather than 500 ps trajectories which considerably reduces the runtime of our simulations. Finally, we would like to preserve the original LIE structure of two parameters in our ESF as for other molecular systems the fitted beta parameter could easily increase again.

The small electrostatic weight obtained here is in contrast to what least-squares fitting in LIE modeling usually returns. However, small fitted electrostatics weights such as in the range of 0.033 (compared to 0.221 for the VDW weight) have already been reported[^11^](https://www.zotero.org/google-docs/?0shzKT). Furthermore, hydrophobicity has been shown to be the major factor stabilizing protein-protein association[^10^](https://www.zotero.org/google-docs/?9G6nQ3). The most obvious explanation for the small electrostatic weight is based on the huge binding interface of the hACE2 ligand comprising around 35 amino acids (ca. 500 atoms) compared to simple protein-ligand interfaces with much smaller ligands and therefore much less noise. This hypothesis is supported by two observations. According to our studies, the variance of electrostatic energies over 50 MD replicates is in the same range as the averaged energy itself which is concomitant with a large uncertainty. And secondly, increasing the hACE2 interface selection cutoff from 5 to 10 Å further reduces weights for electrostatic interactions even to a negative value. In contrast, the more rapidly declining VDW interactions appear to be more stable and significantly less sensitive to small displacements in atom positions[^12^](https://www.zotero.org/google-docs/?KCzp1K), noise and interface size. As a consequence, VDW contributions clearly dominate our binding energy models.

The performance of our favored predictive model (model A.1) as presented above had been tuned in advance by evaluating various simulation time ranges ([50, 100, 200, 500, 1000, 2000] ps) and replicate numbers (10, 20, 30, 40, 50). For each combination of these two optimization parameters, we calculated two sets of binding energies Δ*G*, once by least-squares fitting and once through LOOCV, along with corresponding MAEs and correlation coefficients *R*². According to the last prediction column in Table S1 (visualized in Figure 3a), the highest prediction accuracy and lowest error was achieved with 50 replicates of 200 ps molecular dynamics (MD) simulation trajectories, where, for model fitting and cross-validation respectively, *R*² amounts to 0.77 and 0.74 with an average error of less than 1.9 and 2.0 kJ/mol. In the case of this two-parameter model A.1, the accuracy of 500 ps was close behind, but for other model variations (A.2-A.4) we observed the opposite as 500 ps trajectories performed slightly better than 200 ps. Anyway, keeping model A.1 as a reference, MD simulations longer than 200 ps, in particular 1 and 2 ns trajectories, did not increase the model performance in terms of MAE and *R*² which are, by the way, strongly correlated (*r*^2^ > 0.9). As expected, the highest accuracy was obtained with the highest number (50) of replicates.

| **Time** | **replicates** | **10** | | **20** | | **30** | | **40** | | **50** | |
| --- | --- | --- | --- | --- | --- | --- | --- | --- | --- | --- | --- |
| **[ps]** | **measure** | **fit** | **loo** | **fit** | **loo** | **fit** | **loo** | **fit** | **loo** | **fit** | **loo** |
| **50** | ***R*^2^** | 0.54 | 0.52 | 0.62 | 0.59 | 0.66 | 0.63 | 0.67 | 0.64 | 0.68 | 0.65 |
|  | **MAE** | 2.89 | 03.02 | 2.37 | 2.48 | 2.27 | 2.38 | 2.36 | 2.47 | 2.28 | 2.39 |
| **100** | ***R*^2^** | 0.52 | 0.49 | 0.64 | 0.61 | 0.69 | 0.66 | 0.71 | 0.68 | 0.73 | 0.70 |
|  | **MAE** | 2.87 | 3.00 | 2.24 | 2.35 | 2.15 | 2.25 | 2.16 | 2.27 | 2.07 | 2.17 |
| **200** | ***R*^2^** | 0.57 | 0.54 | 0.66 | 0.63 | 0.73 | 0.70 | 0.74 | 0.72 | **0.77** | **0.74** |
|  | **MAE** | 2.55 | 2.66 | 2.18 | 2.29 | 2.02 | 2.12 | 1.94 | 2.03 | **1.87** | **1.96** |
| **500** | ***R*^2^** | 0.55 | 0.52 | 0.66 | 0.62 | 0.73 | 0.70 | 0.73 | 0.70 | 0.77 | 0.73 |
|  | **MAE** | 2.49 | 2.58 | 2.20 | 2.30 | 02.03 | 2.13 | 2.00 | 2.10 | 1.86 | 1.95 |
| **1000** | ***R*^2^** | 0.63 | 0.58 | 0.59 | 0.56 | 0.59 | 0.55 | 0.66 | 0.62 | 0.69 | 0.65 |
|  | **MAE** | 2.28 | 2.41 | 2.43 | 2.56 | 2.45 | 2.57 | 2.33 | 2.45 | 2.24 | 2.34 |
| **2000** | ***R*^2^** | 0.60 | 0.55 | 0.54 | 0.49 | 0.51 | 0.45 | 0.61 | 0.55 | 0.63 | 0.57 |
|  | **MAE** | 2.34 | 2.48 | 2.58 | 2.72 | 2.66 | 2.79 | 2.57 | 2.69 | 2.48 | 2.60 |

**Table S2:** Coefficients of determination (*R*²) and mean absolute errors (MAE, given in kJ/mol) for training set fitting (fit) and leave-one-out cross-validation (loo) optimized over multiple MD time ranges and replicates. Data generated by two-parameter ESF used in model A.1 (VDW and electrostatics).

| **Time** | **replicates** | **10** | | **20** | | **30** | | **40** | | **50** | |
| --- | --- | --- | --- | --- | --- | --- | --- | --- | --- | --- | --- |
| **[ps]** | **measure** | **fit** | **loo** | **fit** | **loo** | **fit** | **loo** | **fit** | **loo** | **fit** | **loo** |
| **50** | ***R*^2^** | 0.57 | 0.52 | 0.64 | 0.58 | 0.67 | 0.62 | 0.68 | 0.63 | 0.69 | 0.65 |
|  | **MAE** | 2.59 | 2.76 | 2.27 | 2.44 | 2.23 | 2.41 | 2.25 | 2.41 | 2.22 | 2.37 |
| **100** | ***R*^2^** | 0.54 | 0.48 | 0.65 | 0.60 | 0.69 | 0.65 | 0.71 | 0.67 | 0.73 | 0.69 |
|  | **MAE** | 2.59 | 2.76 | 2.22 | 2.38 | 2.15 | 2.32 | 2.13 | 2.29 | 2.06 | 2.21 |
| **200** | ***R*^2^** | 0.58 | 0.53 | 0.66 | 0.62 | 0.73 | 0.69 | 0.75 | 0.71 | 0.77 | 0.74 |
|  | **MAE** | 2.43 | 2.58 | 2.20 | 2.36 | 2.02 | 2.17 | 1.94 | 2.08 | 1.87 | 2.01 |
| **500** | ***R*^2^** | 0.55 | 0.51 | 0.66 | 0.62 | 0.74 | 0.71 | 0.75 | 0.71 | **0.79** | **0.76** |
|  | **MAE** | 2.44 | 2.58 | 2.16 | 2.30 | 1.89 | 2.03 | 1.85 | 1.99 | **1.71** | **1.84** |
| **1000** | ***R*^2^** | 0.63 | 0.58 | 0.60 | 0.54 | 0.59 | 0.54 | 0.67 | 0.63 | 0.71 | 0.67 |
|  | **MAE** | 2.23 | 2.40 | 2.41 | 2.59 | 2.44 | 2.62 | 2.20 | 2.35 | 2.09 | 2.24 |
| **2000** | ***R*^2^** | 0.60 | 0.53 | 0.54 | 0.48 | 0.52 | 0.45 | 0.63 | 0.58 | 0.67 | 0.62 |
|  | **MAE** | 2.34 | 2.54 | 2.58 | 2.78 | 2.60 | 2.79 | 2.31 | 2.48 | 2.18 | 2.34 |

**Table S3:** Coefficients of determination (*R*²) and mean absolute errors (MAE, given in kJ/mol) for training set fitting (fit) and leave-one-out cross-validation (loo) optimized over multiple MD time ranges and replicates. Data generated by two-parameter ESF used in model A.2 (VDW and electrostatics, constant).

| **Time** | **replicates** | **10** | | **20** | | **30** | | **40** | | **50** | |
| --- | --- | --- | --- | --- | --- | --- | --- | --- | --- | --- | --- |
| **[ps]** | **measure** | **fit** | **loo** | **fit** | **loo** | **fit** | **loo** | **fit** | **loo** | **fit** | **loo** |
| **50** | ***R*^2^** | 0.56 | 0.55 | 0.63 | 0.62 | 0.65 | 0.64 | 0.67 | 0.66 | 0.67 | 0.66 |
|  | **MAE** | 3.61 | 3.69 | 2.90 | 2.97 | 03.03 | 3.10 | 2.85 | 2.92 | 2.70 | 2.76 |
| **100** | ***R*^2^** | 0.53 | 0.51 | 0.64 | 0.62 | 0.67 | 0.66 | 0.69 | 0.68 | 0.70 | 0.69 |
|  | **MAE** | 3.39 | 3.47 | 2.62 | 2.69 | 2.82 | 2.88 | 2.57 | 2.64 | 2.42 | 2.48 |
| **200** | ***R*^2^** | 0.57 | 0.56 | 0.64 | 0.63 | 0.69 | 0.67 | 0.72 | 0.71 | 0.73 | 0.72 |
|  | **MAE** | 2.79 | 2.86 | 2.42 | 2.48 | 2.35 | 2.41 | 2.16 | 2.21 | 2.07 | 2.12 |
| **500** | ***R*^2^** | 0.55 | 0.53 | 0.63 | 0.62 | 0.69 | 0.68 | 0.71 | 0.69 | **0.74** | **0.73** |
|  | **MAE** | 2.63 | 2.69 | 2.27 | 2.32 | 2.11 | 2.16 | 2.05 | 2.10 | **1.89** | **1.94** |
| **1000** | ***R*^2^** | 0.62 | 0.61 | 0.59 | 0.58 | 0.57 | 0.55 | 0.63 | 0.61 | 0.67 | 0.65 |
|  | **MAE** | 2.48 | 2.55 | 2.54 | 2.60 | 2.57 | 2.63 | 2.41 | 2.46 | 2.28 | 2.33 |
| **2000** | ***R*^2^** | 0.59 | 0.57 | 0.54 | 0.52 | 0.48 | 0.46 | 0.58 | 0.55 | 0.62 | 0.59 |
|  | **MAE** | 2.38 | 2.44 | 2.59 | 2.65 | 2.72 | 2.79 | 2.59 | 2.65 | 2.48 | 2.54 |

**Table S4:** Coefficients of determination (*R*²) and mean absolute errors (MAE, given in kJ/mol) for training set fitting (fit) and leave-one-out cross-validation (loo) optimized over multiple MD time ranges and replicates. Data generated by two-parameter ESF used in model A.3 (VDW).

| **Variant** | **Experimental affinities Barton *et al.*** | | | **Experimental affinities Han *et al.*** | | | **Experimental affinities Cameroni *et al.*** | | | **ESF** | |
| --- | --- | --- | --- | --- | --- | --- | --- | --- | --- | --- | --- |
|  | ***K*_D_**  **[nM]** | ***K*_D_**  **ratio** | **ΔΔ*G***  **[kJ/mol]** | ***K*_D_**  **[nM]** | ***K*_D_**  **ratio** | **ΔΔ*G***  **[kJ/mol]** | ***K*_D_**  **[nM]** | ***K*_D_**  **ratio** | **ΔΔ*G***  **[kJ/mol]** | ***K*_D_**  **ratio** | **ΔΔ*G***  **[kJ/mol]** |
| **WT** | 74.40 | 1.00 | 0.00 | 24.63 | 1.00 | 0.00 | 60.00 | 1.00 | 0.00 | 1.00 | 0.0 |
| **Alpha** | 7.00 | 0.09 | -6.09 | 5.40 | 0.22 | -3.91 | 9.60 | 0.16 | -4.72 | 0.07 | -6.7 |
| **Beta** | 20.00 | 0.27 | -3.39 | 13.83 | 0.56 | -1.49 | 25.10 | 0.42 | -2.25 | 0.56 | -1.5 |
| **Gamma** | 13.50 | 0.18 | -4.40 | 11.00 | 0.45 | -2.08 |  | *n.d.* |  | 0.21 | -4.0 |
| **Delta** | *n.d.* | | | 25.07 | 1.02 | 0.05 | 75.00 | 1.25 | 0.58 | 0.69 | -0.9 |
| **Delta plus** | *n.d.* | | |  | *n.d.* |  |  | *n.d.* |  | 0.42 | -2.2 |
| **Omicron** | *n.d.* | | | 31.40 | 1.27 | 0.63 | 25.30 | 0.42 | -2.23 | 0.13 | -5.2 |
| ***R*²**  **MAE** |  |  | 0.87  0.73 |  |  | 0.28  2.14 |  |  | 0.63  1.72 |  |  |

**Table S5.** SARS-CoV-2 VOC RBDs relative binding free energies (ΔΔ*G*) and ratios of dissociation constants in relation to the wild type RBD predicted by our empirical scoring function compared to results determined through surface plasmon resonance by Barton *et al.*[^15^](https://www.zotero.org/google-docs/?TEr1dg), Han *et al.*[^13^](https://www.zotero.org/google-docs/?tRcai8) and Cameroni *et al.*[^14^](https://www.zotero.org/google-docs/?haNic0) for available VOCs. Coefficients of determination (*R*²) and mean absolute errors (MAE, given in kJ/mol) for experimental ΔΔ*G*’s to predicted ΔΔ*G*’s are given below.

**References**

[1. Gruber, K., Steinkellner, G. & Gruber, C. Determining novel enzymatic functionalities using three-dimensional point clouds representing physico chemical properties of protein cavities, WO2014080005A1. (2020).](https://www.zotero.org/google-docs/?aT27QZ)

[2. Adhya, L., Mapder, T. & Adhya, S. Role of terminal dipole charges in aggregation of α-helix pair in the voltage gated K+ channel. *Biochim. Biophys. Acta BBA - Biomembr.* **1828**, 845–850 (2013).](https://www.zotero.org/google-docs/?aT27QZ)

[3. Hol, W. G. J., Halie, L. M. & Sander, C. Dipoles of the α-helix and β-sheet: their role in protein folding. *Nature* **294**, 532–536 (1981).](https://www.zotero.org/google-docs/?aT27QZ)

[4. Sengupta, D., Behera, R. N., Smith, J. C. & Ullmann, G. M. The α Helix Dipole: Screened Out? *Structure* **13**, 849–855 (2005).](https://www.zotero.org/google-docs/?aT27QZ)

[5. Hendlich, M., Rippmann, F. & Barnickel, G. LIGSITE: automatic and efficient detection of potential small molecule-binding sites in proteins. *J. Mol. Graph. Model.* **15**, 359–363 (1997).](https://www.zotero.org/google-docs/?aT27QZ)

[6. Steinkellner, G. *et al.* Identification of promiscuous ene-reductase activity by mining structural databases using active site constellations. *Nat. Commun.* **5**, 1–9 (2014).](https://www.zotero.org/google-docs/?aT27QZ)

[7. Prattes, M. *et al.* Structural basis for inhibition of the AAA-ATPase Drg1 by diazaborine. *Nat. Commun.* **12**, 3483 (2021).](https://www.zotero.org/google-docs/?aT27QZ)

[8. Aqvist, J., Medina, C. & Samuelsson, J. E. A new method for predicting binding affinity in computer-aided drug design. *Protein Eng.* **7**, 385–391 (1994).](https://www.zotero.org/google-docs/?aT27QZ)

[9. Hansson, T. & Aqvist, J. Estimation of binding free energies for HIV proteinase inhibitors by molecular dynamics simulations. *Protein Eng.* **8**, 1137–1144 (1995).](https://www.zotero.org/google-docs/?aT27QZ)

[10. Chothia, C. & Janin, J. Principles of protein–protein recognition. *Nature* **256**, 705–708 (1975).](https://www.zotero.org/google-docs/?aT27QZ)

[11. Pereira, E. G., Moreira, M. A. M. & Caffarena, E. R. Molecular interactions of c-ABL mutants in complex with imatinib/nilotinib: a computational study using linear interaction energy (LIE) calculations. *J. Mol. Model.* **18**, 4333–4341 (2012).](https://www.zotero.org/google-docs/?aT27QZ)

[12. Singh, A., Steinkellner, G., Köchl, K., Gruber, K. & Gruber, C. C. Serine 477 plays a crucial role in the interaction of the SARS-CoV-2 spike protein with the human receptor ACE2. *Sci. Rep.* **11**, 4320 (2021).](https://www.zotero.org/google-docs/?aT27QZ)

[13. Han, P. *et al.* Receptor binding and complex structures of human ACE2 to spike RBD from omicron and delta SARS-CoV-2. *Cell* **185**, 630-640.e10 (2022).](https://www.zotero.org/google-docs/?aT27QZ)

[14. Cameroni, E. *et al.* Broadly neutralizing antibodies overcome SARS-CoV-2 Omicron antigenic shift. *Nature* **602**, 664–670 (2022).](https://www.zotero.org/google-docs/?aT27QZ)

[15. Barton, M. I. *et al.* Effects of common mutations in the SARS-CoV-2 Spike RBD and its ligand, the human ACE2 receptor on binding affinity and kinetics. *eLife* **10**, e70658.](https://www.zotero.org/google-docs/?aT27QZ)
